# Boundless love: a German Professor Uli Schwarz in China

**DOI:** 10.1093/procel/pwae019

**Published:** 2024-04-22

**Authors:** Le Kang

**Affiliations:** State Key Laboratory of Integrated Management of Pest Insects and Rodents, Institute of Zoology, Chinese Academy of Sciences, Beijing 100101, China

More than 20 years ago, an old German man embarked on a quest to explore the suburbs of Shanghai and the villages of Jiangsu Province in search of antique Chinese furniture. He was not a mere antique collector or aficionado of ancient furniture, but a German scientist motivated by his profound love of Chinese culture. His name was Uli Schwarz (Fig. 1), a distinguished German scientist who had dedicated the majority of his life to promoting cross-cultural scientific exchanges, an effort that left an indelible mark on the landscape of research collaborations between China and Germany.

**Figure 1.**
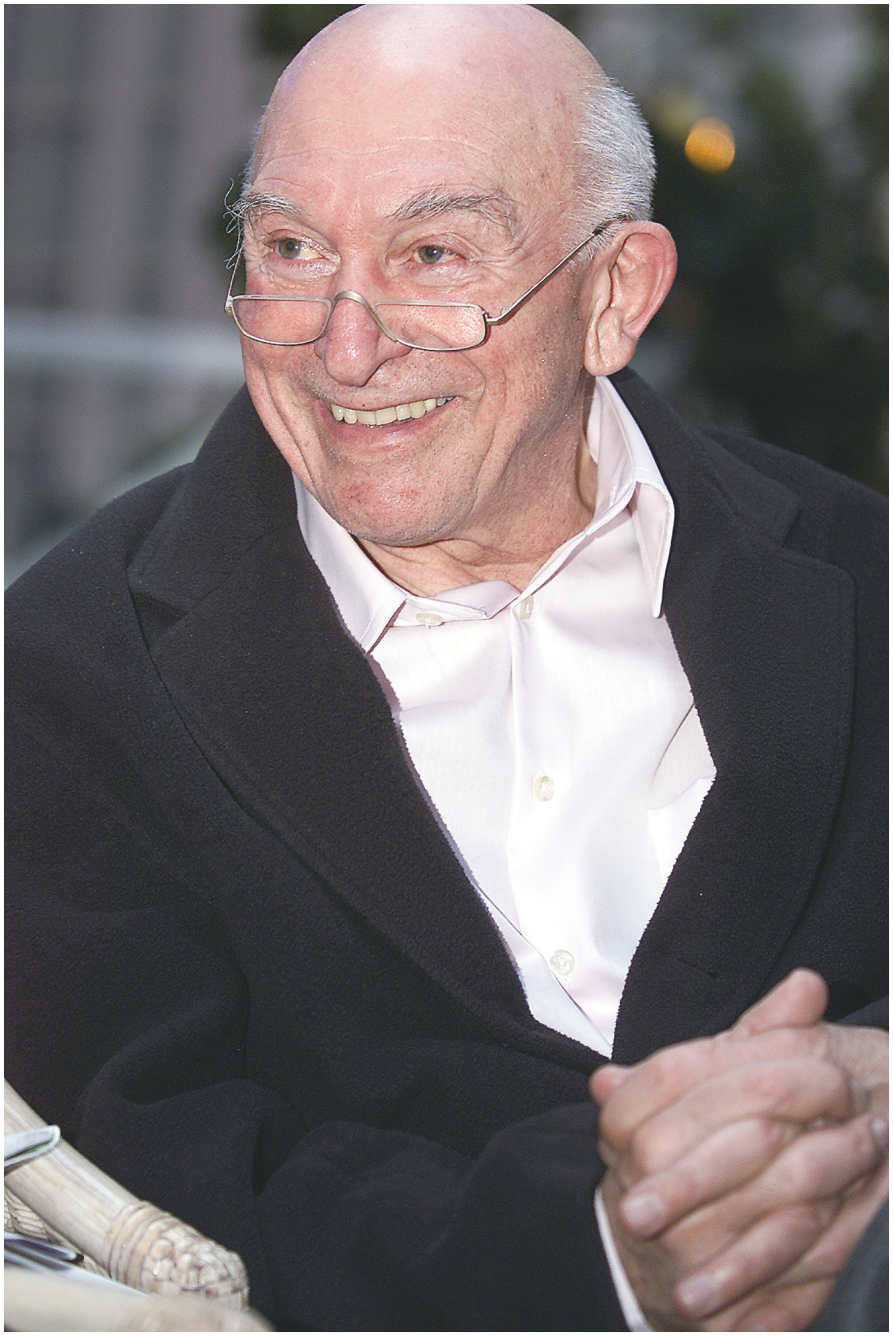
Prof. Uli Schwarz (1934–2006).

My initial encounter with Prof. Uli Schwarz occurred in 1999 when I served as a leader of the Bureau of Life Science and Biotechnology, Chinese Academy of Sciences (CAS). I participated in a special meeting for the organization of an Independent Max Planck Society (MPG) Junior Research Group in Shanghai. Our initial interaction led to a long-standing collaboration, during which Prof. Schwarz and I co-organized several conferences and nurtured life-science collaborations between CAS and MPG. His vast knowledge and authentic character left an indelible impression on me, and the experience remains one of my most cherished memories.

Prof. Schwarz considered Shanghai to be his home in China, where he lived in a French-style villa (Fig. 2) built in 1928, adorned with Chinese antique furniture that he had collected. This architectural marvel, situated in the scenic gardens of the Shanghai Branch of CAS, served as a scientific communication hub and evolved into the Shanghai Institute for Advanced Studies (SIAS). Now, it has become the academic base of the Shanghai Branch of CAS. Over the years, this center has hosted numerous scientists from various parts of the world, facilitating round-table discussions and fostering the international collaborations, which Prof. Schwarz tirelessly advocated. Regrettably, in 2006, Prof. Uli Schwarz passed away from a heart attack in Germany, leaving behind a long legacy of fostering German–Chinese scientific collaborations. In SIAS, in this beautiful French-style villa, in its elegant meeting room, dining rooms, and living spaces decorated with Chinese furniture (Fig. 3), in which Western architecture is blended with Chinese furniture, Uli Schwarz’s vision continues to burn brightly in the 15 years since his departure.

**Figure 2.**
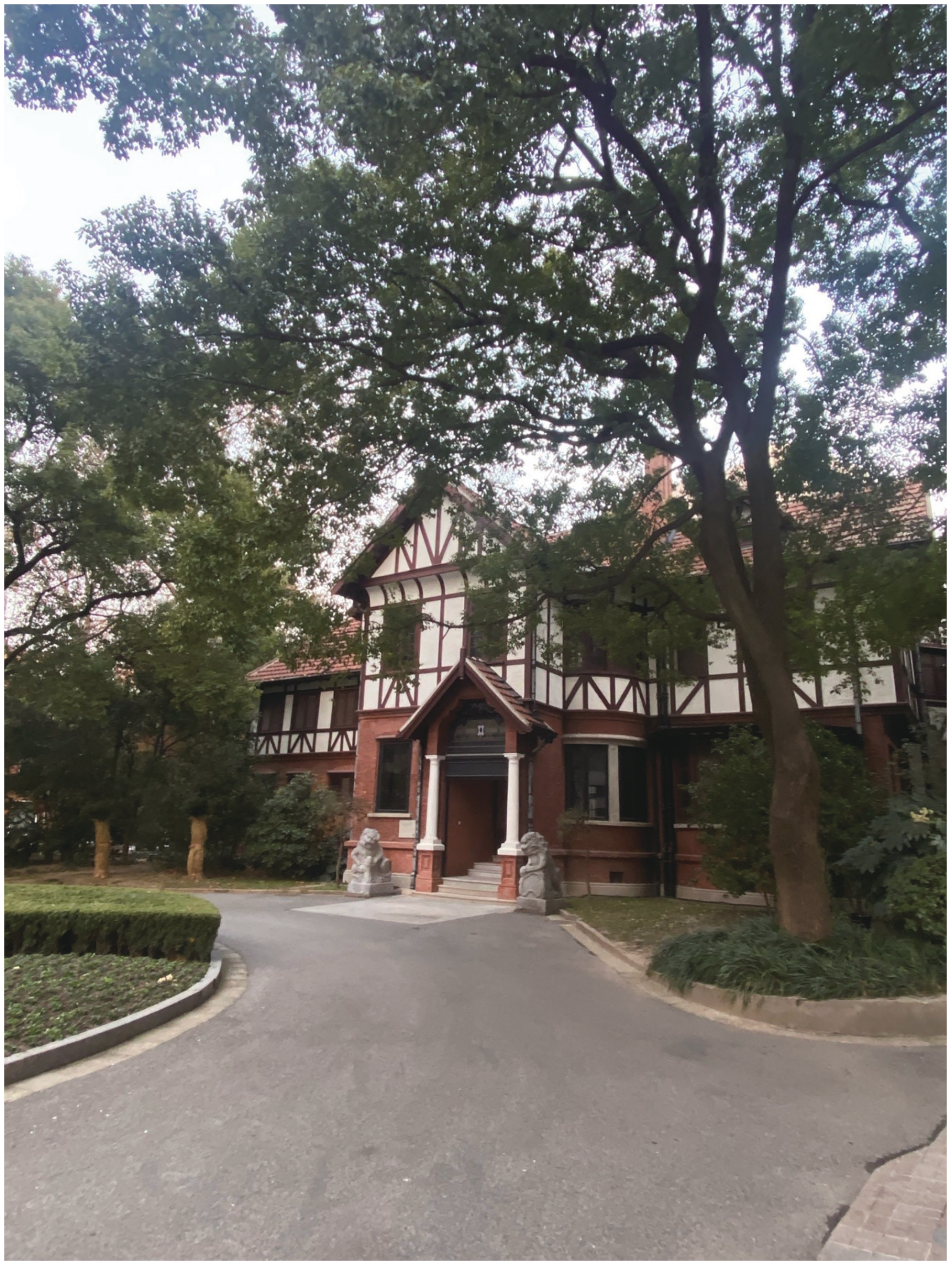
Shanghai Institute for Advanced Studies (SIAS).

**Figure 3.**
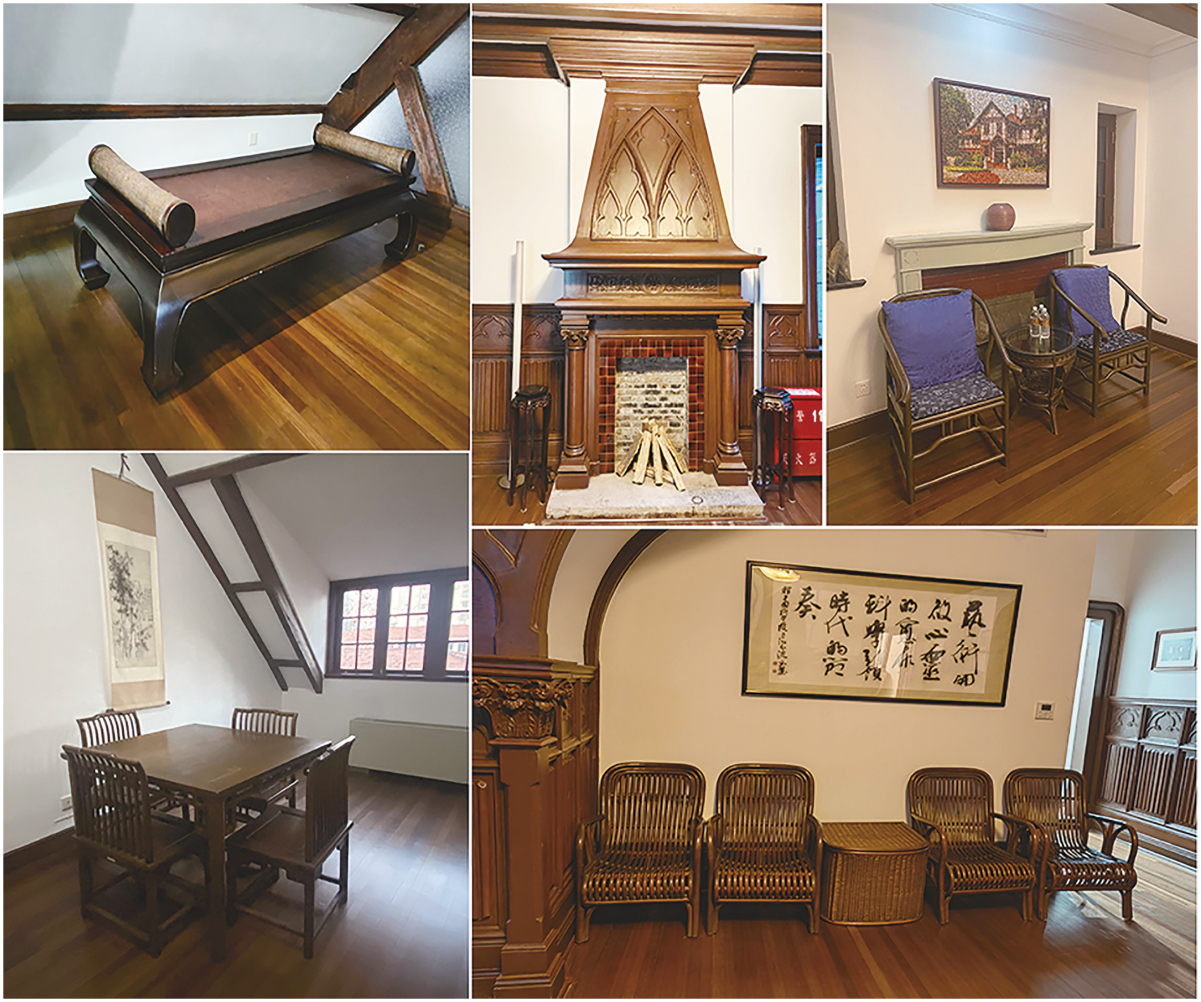
Chinese furniture in SIAS.

Prof. Uli Schwarz immersed himself deeply in Chinese society and harbored a profound fondness for China. From 1978 to 2006, he tirelessly traveled above 100 times between China and Germany and led a number of joint research initiatives, exchange programs, and collaborative projects that propelled Chinese scientific research onto the global stage. Under his stewardship, numerous breakthroughs were achieved, fostering an academic environment of innovation and excellence in China. In recognition of his contributions, he was honored with the Medal of the People’s Republic of China for International Cooperation in Science and Technology in 1997, and the Friendship Award of the People’s Republic of China in 2003 (Fig. 4).

**Figure 4.**
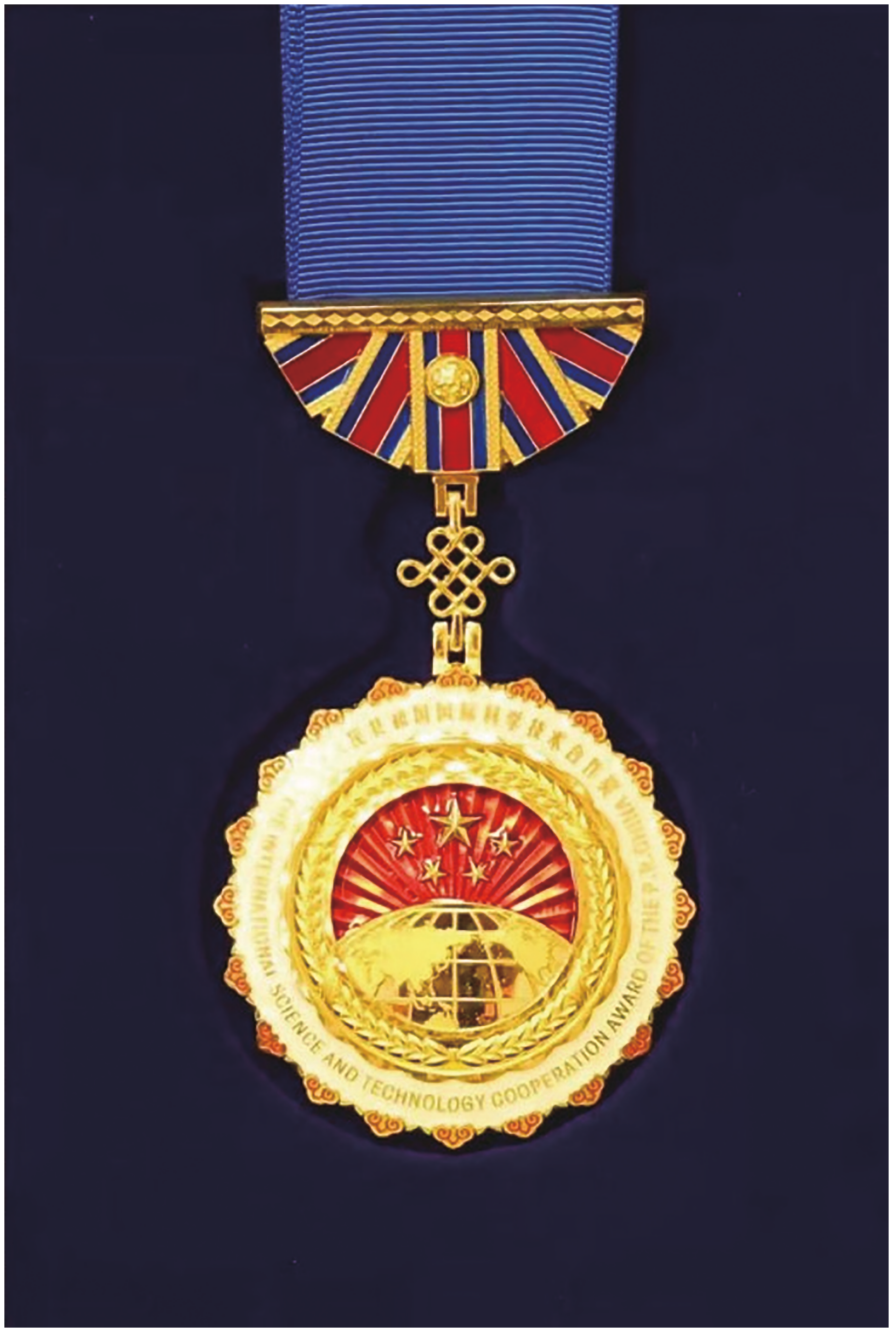
Prof. Schwarz was awarded with Friendship Award of the People’s Republic of China in 2003.

Prof. Uli Schwarz played an exceptional role as a bridge between the CAS and the MPG, facilitating a seamless exchange of knowledge and expertise between these two nations both with rich traditions in research and innovation. His efforts have not only advanced scientific frontiers but also deepened mutual understanding and appreciation, transcending geographical, and cultural boundaries.

## Proposing the MPG Guest Laboratory

In 1978, a pivotal cooperation agreement was signed between the CAS and MGP to facilitate scientific exchanges and enhance collaboration between institutions that could advance Chinese scientific development. However, the disparity in scientific development between China and Germany made many German scientists apprehensive of long-term research engagements in China. However, a deep professional friendship was able to overcome these reservations.

In 1981, Prof. Uli Schwarz joined forces with Prof. Xiaohui Zhuang, a famous experimental embryologist and director of the Institute of Cell Biology of CAS, to establish an open laboratory in China. Prof. Zhuang had received his doctorate from the University of Munich in 1939, returning to China in 1946, and their friendship had grown over the years. Four years later, on the occasion of the 10th anniversary of the CAS–MPG cooperation, the MPG Guest Laboratory was inaugurated at the Institute of Cell Biology of CAS in Shanghai. The laboratory introduced advanced concepts of international organization of scientific activities, enabling overseas Chinese students to continue their studies initiated abroad upon their return, and served as a platform for scientists to conduct experiments and educate young scientists and graduate students. This support, in conjunction with the existing cooperation, facilitated the visits of over 100 scientists from MPG and other European universities to China. During these visits, experiments were conducted alongside Chinese colleagues, cutting-edge academic lectures were delivered and students were mentored in various fields such as cell, molecular, and developmental biology, and neuro biology.

## Organization of the independent MPG Junior Research Group

**Figure 5.**
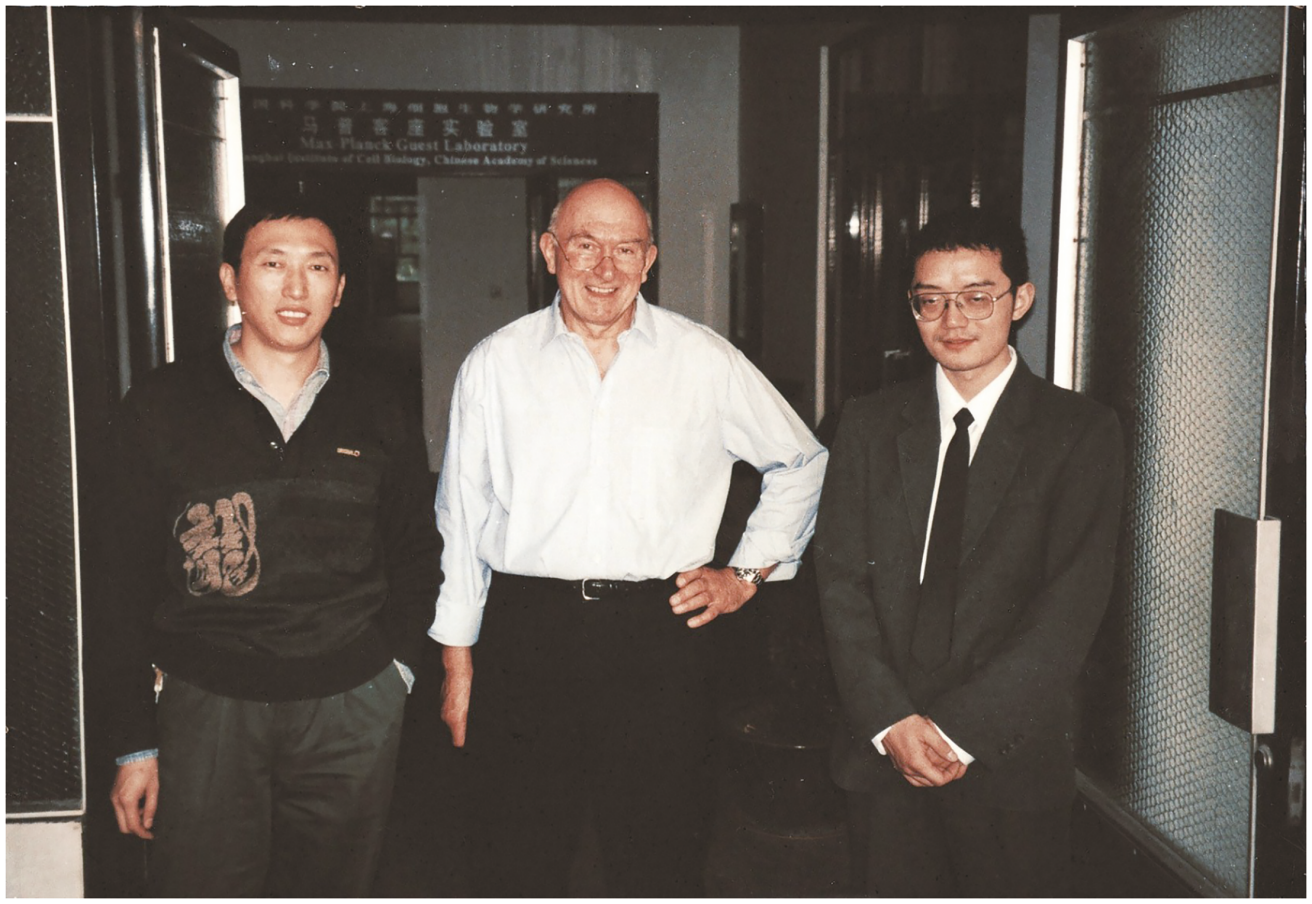
Prof. Schwarz and leaders of the Independent Max Planck Junior Research Group (left: Gang Pei; right: Gengxi Hu).

In 1995, Prof. Uli Schwarz proposed to transplant to China the MPG's successful experience of selecting, training, and empowering young scientific leaders. In pursuit of this vision, he advocated for the establishment of an Independent Max Planck Junior Research Group in Shanghai. The primary objective was to attract Chinese talent from abroad to return to China and foster Chinese interdisciplinary communication and scientific innovation. The initiative convinced a cohort of young Chinese researchers residing overseas to return home and pursue their research careers in China. Focusing on advancing China’s scientific development, Prof. Uli Schwarz prioritized the selection of Chinese young scientists for leadership position within the group. After a rigorous screening process, three young Chinese doctors emerged as formal candidates in 1995, with Prof. Gang Pei ultimately selected as the inaugural leader of the Independent Max Planck Junior Research Group (Fig. 5). Subsequently, Prof. Gang Pei was elected a Member of the CAS and appointed as the director of the Shanghai Institutes for Biological Sciences (SIBS) 4 years later. In 2008, he was appointed president of Tongji University by the China Ministry of Education. When participated in Uli Schwarz’s funeral, Prof. Pei fondly recollected his best friend and confidant, offering high praise for Prof. Uli Schwarz and noting that he embodied all the qualities that a traditional Chinese gentleman should have: “Ren” “Yi” “Li” “Zhi” “Xin” (in English: benevolence, righteousness, courtesy, wisdom, and trust) (Pei, 2007).

Beyond the realms of research, Prof. Uli Schwarz emerged as a stalwart advocate for the development of effective management formats within the scientific community in China. The founding of the Independent MPG Junior Research Group was groundbreaking, playing a pivotal role in reforming China’s academic management system. The successful experiment in Shanghai encouraged Prof. Schwarz to expand the program of Junior Research Group Leaders to universities and research institutes in Beijing, Wuhan, and Kunming. His contributions in this domain have not only helped China to keep pace in the fiercely competitive world of cutting-edge research but have also equipped the nation with the necessary tools for sustainable advancement and effective governance.

## Establishment of the Shanghai Institute for Advanced Studies (SIAS)

In 2002, Prof. Uli Schwarz co-founded the Shanghai Institute for Advanced Studies with Prof. Yi Rao, creating a platform to foster dialogue among international scientists and strategize research initiatives. This kind of center was initially established at Princeton University, USA, with the aim of facilitating global cooperation and stimulating the development of cutting-edge fields and interdisciplinary directions. Not wanting to simply replicate existing institutions in the Western countries, Prof. Uli Schwarz stressed that the SIAS had to establish its own path to achieve its goals. This strategic orientation underscored Prof. Uli Schwarz’s dedication to fostering Chinese innovation and advancing the frontiers of scientific inquiry. He emphasized the pivotal role of scientists in shaping the future by developing new theoretical hypotheses and concepts conducive to economic and societal development in response to evolving environmental and technological advancements.

**Figure 6.**
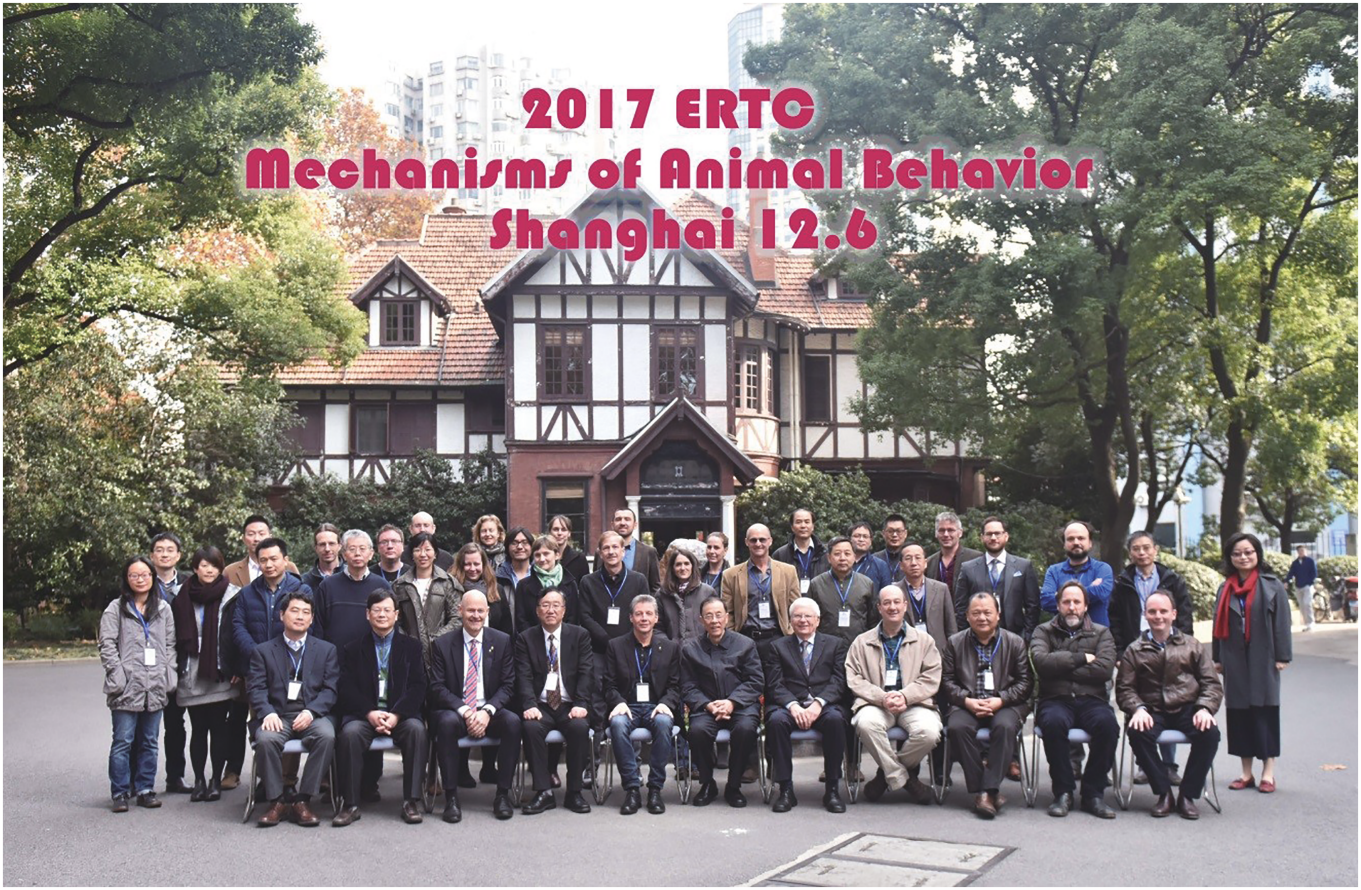
Exploratory Round Table Conference on Mechanism of Animal Behavior in 2017.

Under the guidance of Prof. Uli Schwarz, SIAS has hosted a variety of events that embraced Chinese culture, including round-tables and science salons, in order to create an open academic environment and advance cooperation and innovation in China. The specific objectives of these round-tables included topics such as “High Technology in Ancient China: Sources, Achievements, and Admonitions,” “Explorations and Perspectives on the Biology of Infectious Diseases,” and “The Ecology of Big Cities,” underscore Prof. Schwarz’s strategic vision and his deep interest in Chinese society. In addition, Prof. Uli Schwartz was keenly aware of the reality of scientific and technological development in China and its specific needs. To this end, he organized an interdisciplinary seminar in Shanghai to discuss prospective concepts and novel directions related to the theme of “Scientific Emotions” and “the Chinese Role in the International Community in the New Century.” Under this platform, Dr. Manfred Gahr and I also had the pleasure of organizing an Exploratory Round Table Conference on the Mechanism of Animal Behavior in 2017 (Fig. 6).

In 2003, with the help of two junior group leaders, Zhendong Dai (Nanjing University of Astronautics and Aeronautics) and Stanislav Gorb (from his lab in Tübingen, Germany), he organized the first international conference on Biomimetics/Bionics, which was not only a great start for new science and technology fields in China but also a long-term fruitful collaboration between Chinese and German scientists on bioinspiration, surface science, and robotics, fields which were actually far from his own expertise. Meanwhile, the International Society of Bionics Engineering with the main headquarter situated at Jilin University, China counts over 4,800 members worldwide. This fact shows an exceptional talent of Prof. Uli Schwarz in identifying novel promising scientific fields and at the same time in identifying and supporting talented young persons, who could take these fields to new heights. Collaboration established in 2003 between Prof. Dai and Prof. Gorb is flourishing today as 20 years before.

## Efforts to establish the CAS–MPG Partner Institute for Computational Biology

At the turn of the century, the leadership of the Shanghai Institute for Advanced Studies (SIAS) recognized the growing significance of computational biology and bioinformatics in life sciences research. During the fourth round-table discussion, the concept of establishing a computational biology institute in China was introduced. Prof. Uli Schwarz, who was approaching 70 years at the time, was wholeheartedly committed to realizing this vision. In an effort to substantiate the feasibility of establishing the institute, the SIAS organized two strategic seminars on “Prospects for Computational Biology” in 2004. Subsequently, on November 8, Prof. Yongxiang Lu and Prof. Peter Gruss signed a cooperation agreement and the institute’s statute, marking a pivotal moment in the collaboration between the two institutions. I have visited the headquarters of the Max Plank Society in Germany three times for the implementation of this cooperation. In 2005, CAS–MPG Partner Institute for Computational Biology was officially established in Shanghai. Dr Li Jin, now the president of Fudan University, was appointed as the institute’s Founding Director from China’s side. Notably, the joint institute stands as the first physical research institute of the MPG to be co-established outside of Germany. In 2006, Prof. Uli Schwarz passed away due to a heart attack in Germany. However, this tragic event did not interrupt the normal functioning of the institute. What he left behind was not merely a collection of scientific achievements but an enduring friendship and ongoing bilateral cooperation between China and Germany. In 2008, the CAS–MPG Partner Institute for Computational Biology, integrated with the Guest Laboratory, was renamed as the “Uli Schwarz Public Experimental Platform” in memory of Prof. Uli Schwarz, acknowledging his substantial contributions to the scientific community in China.

## A sincere German friend with an international strategic vision

Amid challenging times, Prof. Uli Schwarz demonstrated exceptional efforts to promote Chinese talent acquisition and drive Science and Technology (S&T) reform, drawing on profound scientific expertise and strategic insight into Chinese scientific development. His involvement led to significant milestones in the growth of Chinese scientific research across four academic institutes. Prof. Peter Gruss underscored the pivotal role of the Shanghai Institute for Advanced Studies as a crucial hub of Chinese influence on the global stage within a dynamic and ever-evolving environment. Prof. Uli Schwarz placed great value on shaping the next generation of scientists and leaders in China. He emphasized the significance of nurturing a passion for inquiry and encouraged their participation in communication for scientific inspiration. To this end, he organized various international seminars and workshops, consistently inviting young Chinese researchers to join renowned scientists from abroad. The vivid memories of the friendly and open atmosphere during the discussions at these symposium endure for the participants.

During his 30-year tenure in China, Prof. Uli Schwarz skillfully integrated Chinese and Western cultures into both his personal life and research endeavors, establishing a profound emotional connection with China. On each visit to China, he made a point to visit the homes of his Chinese friends and brought with him small gifts representative of Western culture. In August 2006, during his participation in the International Symposium on Primate Biomedicine in Kunming, China, little did anyone anticipate that this would mark his final visit to China, which he regarded as his second home. Notably, during this trip, Prof. Uli Schwarz donated 80,000 yuan from his personal savings to the Hongwen Village Primary School in Lijiang, Yunnan Province, to support the repair of the school building and the purchase of essential school supplies. Sadly, this ongoing contribution was abruptly halted in December 2006, following his passing. However, his impact in promoting Sino-German scientific and technological exchanges endures, owing to his embodiment of sincerity and selflessness, akin to a gentle wind that gives without expecting anything in return.

In the cold winter of 2023, as I revisited the Shanghai Institute for Advanced Studies (SIAS), the French villa, surrounded by verdant trees, stood resolute against the biting wind. Though the physical landscape remained unchanged, the prevailing essence has evolved. It is evident that the enduring bonds of camaraderie initiated by Prof. Uli Schwarz will continue to flourish and mature, akin to the growth of SIAS’s sheltering trees.
